# Equine CTNNB1 and PECAM1 nucleotide structure and expression analyses in an experimental model of normal and pathological wound repair

**DOI:** 10.1186/1472-6793-8-1

**Published:** 2008-01-31

**Authors:** Vincenzo Miragliotta, Zoë Ipiña, Josiane Lefebvre-Lavoie, Jacques G Lussier, Christine L Theoret

**Affiliations:** 1 Département de biomédecine vétérinaire, Faculté de médecine vétérinaire, Université de Montréal, C.P. 5000, St-Hyacinthe, Québec, J2S 7C6, Canada; 2 Department of veterinary anatomy, biochemistry and physiology, University of Pisa, Viale delle Piagge 2 56100 Pisa, Italy

## Abstract

**Background:**

Wound healing in horses is fraught with complications. Specifically, wounds on horse limbs often develop exuberant granulation tissue which behaves clinically like a benign tumor and resembles the human keloid in that the evolving scar is trapped in the proliferative phase of repair, leading to fibrosis. Clues gained from the study of over-scarring in horses should eventually lead to new insights into how to prevent unwanted scar formation in humans. cDNA fragments corresponding to *CTNNB1 *(coding for β-catenin) and *PECAM1*, genes potentially contributing to the proliferative phase of repair, were previously identified in a mRNA expression study as being up-regulated in 7 day wound biopsies from horses. The aim of the present study was to clone full-length equine *CTNNB1 *and *PECAM1 *cDNAs and to study the spatio-temporal expression of mRNAs and corresponding proteins during repair of body and limb wounds in a horse model.

**Results:**

The temporal pattern of the two genes was similar; except for *CTNNB1 *in limb wounds, wounding caused up-regulation of mRNA which did not return to baseline by the end of the study. Relative over-expression of both *CTNNB1 *and *PECAM1 *mRNA was noted in body wounds compared to limb wounds. Immunostaining for both β-catenin and PECAM1 was principally observed in endothelial cells and fibroblasts and was especially pronounced in wounds having developed exuberant granulation tissue.

**Conclusion:**

This study is the first to characterize equine cDNA for *CTNNB1 *and *PECAM1 *and to document that these genes are expressed during wound repair in horses. It appears that β-catenin may be regulated in a post-transcriptional manner while PECAM1 might help thoracic wounds mount an efficient inflammatory response in contrast to what is observed in limb wounds. Furthermore, data from this study suggest that β-catenin and PECAM1 might interact to modulate endothelial cell and fibroblast proliferation during wound repair in the horse.

## Background

Repair of wounds located on the limbs, but not the body, of horses is often accompanied by the formation of exuberant granulation tissue (EGT) which behaves clinically like a benign tumor and resembles the human keloid [1,2] in that the evolving scar is trapped in the proliferative phase of repair, leading to fibrosis. This condition ultimately generates extensive scarring, detrimental to function. Indeed, a recent study reported that approximately 7% of injuries leading to the retirement of racehorses are the result of a wound [3]. Efforts to enhance wound repair and to prevent unwanted scar formation in both humans and horses have been disappointing, possibly owing to insufficient knowledge of the underlying mechanisms.

We recently identified genes expressed in wound margin biopsies of horses, using suppression subtractive hybridization (SSH) [4]. Two hundred and twenty six cDNAs were found to be differentially expressed during the proliferative phase of repair of normal body wounds, 129 of which matched against GenBank databases. In the study described herein, we targeted a previously identified SSH-derived cDNA fragment corresponding to β-catenin (*CTNNB1*) and a second corresponding to equine platelet endothelial adhesion molecule-1 (*PECAM1*) for their potential contribution to the proliferative phase of repair, which is aberrant following wounding on the limbs of horses. *CTNNB1 *had already been demonstrated to play a role in fibroproliferative disorders [5,6] and *PECAM1 *was chosen because of its known interaction with β-catenin [7].

Beta-catenin is an 88-kDa highly conserved protein that is a structural component of the adherens junction (AJ), a cadherin-dependent adhesive structure intricately linked to the actin microfilament network and located between adjacent epithelial cells. Beta-catenin is a central mediator in the canonical wingless (Wnt) signaling pathway, which exerts remarkable control over cellular phenotype and behavior [8]. When the Wnt pathway is quiescent, β-catenin participates in AJs which must disassemble to allow for cell migration, a process critical to epithelialization following wounding. When freed from intercellular contacts, β-catenin is phosphorylated and targeted for degradation [6]. Activation of the Wnt pathway inhibits phosphorylation, leading to cytosolic stabilization of β-catenin. This stabilized form subsequently translocates to the nucleus where it functions as a transcriptional co-activator of T cell factor/lymphoid enhancer factor (Tcf/Lef) target genes [9].

Beta-catenin plays a cell-specific role during wound repair. Protein levels are transiently elevated in mesenchymal cells during the proliferative phase of repair [5], which is thought to regulate the growth of dermal fibroblasts [10,11]. Moreover, β-catenin stimulates the growth of microvascular endothelial cells [12]. In contrast, β-catenin inhibits migration of human epithelial cells *in vitro*, which may impair wound closure *in vivo *[13]. Hyperplastic wounds in man exhibit a prolonged phase of elevated β-catenin protein levels and concurrent tcf-dependent transcriptional activation of target genes involved in fibroproliferative processes, such as alpha smooth muscle actin (α-SMA), fibronectin (FN) and collagen type III (COLIII) [5]. A transgenic mouse model, in which stabilized β-catenin is expressed in mesenchymal cells, develops aggressive fibromatosis (desmoid tumors) and hyperplastic cutaneous wounds [11].

PECAM1 (CD31) is a 130-kDa transmembrane glycoprotein member of the immunoglobulin superfamily that is constitutively expressed on platelets, specific cells of the immune system, as well as on endothelial cells, particularly at intercellular junctions [14]. Its ectodomain mediates adhesion [15] while its cytoplasmic portion serves as a scaffold for cytoskeletal proteins [16] and for signaling [17]. PECAM1 has been implicated in a number of important biological processes including modulation of adhesion [18], cell migration [19], inflammatory cell infiltration [20,21], phagocytosis by macrophages [22], endothelial permeability [23] and angiogenesis [24,25].

PECAM1 binds tyrosine-phosphorylated β-catenin having left AJs and become localized to the cytosol [16]. Precisely, PECAM1 functions as a reservoir for tyrosine-phosphorylated β-catenin, maintaining localization at the plasma membrane. This dynamic interaction is involved in the proliferation phase of angiogenesis [16]. Biswas et al. [7] identified PECAM1 as a modulator of microvascular endothelial cell proliferation via its participation in the Wnt signaling pathway, thereby stabilizing and promoting the accumulation of transcriptionally active β-catenin.

In the present study we hypothesized that the temporal expression pattern of these two genes with potential roles in angiogenesis and cell proliferation would differ between body wounds which heal normally and limb wounds which are predisposed to the formation of EGT in horses. The specific objectives of this study were to characterize the full-length equine *CTNNB1 *and *PECAM1 *cDNAs, and to study the spatio-temporal expression profile of their mRNAs and proteins during the repair of body and limb wounds. The ultimate objective of our research program is to contribute to a better understanding of the dermal repair process, and permit the development of novel diagnostic and therapeutic strategies to resolve wound healing complications in the horse and which could ultimately be extrapolated to man.

## Results

### Cloning and characterization of equine cDNA for *CTNNB1 *and *PECAM1*

The cDNA fragments used for the virtual Northern analyses came from sequences previously obtained from a gene expression profiling experiment using SSH screening aimed at identifying mRNAs that were increased or induced during the proliferative phase of wound repair in the horse [4].

Screening of the size-selected library from equine thoracic wound margin biopsies collected 7d post-operatively for *CTNNB1 *cDNA resulted in the cloning of a truncated cDNA fragment. Thus, a PCR reaction was performed to characterize 5' upstream sequences of the ORF. The equine *CTNNB1 *cDNA characterized consisted of 2382 bp [GenBank:DQ267491] that included a partial 5'-untranslated region (UTR) of 9 bp, an ORF of 2346 bp encoding a 781-amino acid protein with a theoretical molecular weight of 85.5-kDa, an isoelectric point of 5.5, and a partial 3'-UTR of 27 bp. Amino acid homology search in GenBank by PsiBlast revealed orthologous proteins with an overall identity level of 100% for porcine [GenBank:NM_214367], and 99% to human [GenBank:NM_001904] and canine [GenBank:XM_856013] CTNNB1 proteins. The analysis against a protein conserved domains database (PROSITE) showed the presence of 9 Armadillo/plakoglobin ARM repeat profiles (R^151^-S^191^, Q^193^-G^236^, G^235^-G^277^, G^277^-G^319^, G^319^-G^362^, G^400^-G^442^, G^442^-A^484^, Y^489^-A^532^, N^594^-A^636^).

The virtual Northern analyses allowed determination of the approximate molecular weight of the full-length *PECAM1 *cDNA, which corresponded to 3.1–3.7 kb. The SSH cDNA fragment was thus used as probe to screen, by hybridization, the appropriate size-selected cDNA library generated from equine thoracic wound margin biopsies collected 7d post-operatively. The full-length equine *PECAM1 *cDNA was cloned and consisted of 3381 bp [GenBank:DQ310372] that included a 5'-untranslated region (UTR) of 156 bp, an ORF of 2217 bp encoding a 738-amino acid protein with a theoretical molecular weight of 82.3-kDa, an isoelectric point of 8.2, and a 3'-UTR of 1008 bp containing one polyadenylation signal. Amino acid homology search in GenBank by PsiBlast revealed orthologous proteins with an overall identity level of 76% to canine [GenBank:XM_848326], 75% to porcine [GenBank:NM_213907], and 72% to human [GenBank:NM_000442] and bovine [GenBank:NM_174571] proteins. The equine sequence determined herein contains the 16 exons present in the predominant form of full-length human PECAM1 [26]. While our study does not exclude the possibility of PECAM1 isoforms in the horse, in normal skin and wound edge biopsies we detected only the full-length isoform for PECAM1.

### Temporal expression of *CTNNB1 *and *PECAM1 *mRNAs in body and limb wounds

**Figure 1:**
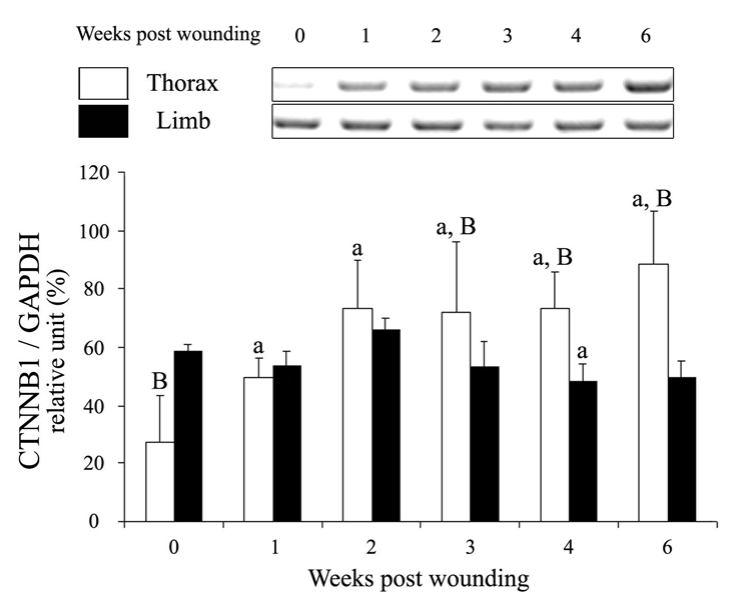
Regulation of equine *CTNNB1 *mRNA by wounding of thorax and limb skin. Total RNA was extracted from wound margin biopsies isolated 1, 2, 3, 4 and 6 weeks post-wounding, then used in mRNA expression analyses by semi-quantitative RT-PCR as described in Methodology. Bar graphs represent the average of measures, performed in triplicate, on the mRNA of the four horses included in the study. Top: Regulation of *CTNNB1 *mRNA (AF 752 bp) in wound biopsies from the thorax and the limb. Bottom: Relative changes in *CTNNB1 *mRNA in biopsies of thorax and limb wounds. The intensity of *CTNNB1 *signals was normalized with the control gene *GAPDH*. Different letters denote samples that differed significantly (P < 0.05) from time 0 of the same site (a); between anatomic sites at the same time (B). Data are presented as means ± SEM.

Semi-quantitative RT-PCR showed an induction of *CTNNB1 *mRNA expression in response to wounding though up-regulation was less acute than for *PECAM1*. The repeated measures linear model revealed a non-significant effect of site, across time (P = 0.49) when used to analyze the temporal expression of *CTNNB1 *mRNA during wound repair in the horse, though constitutive expression of *CTNNB1 *in unwounded skin at time 0 was significantly greater in the limb than in the thorax (P = 0.0014; Fig 1). Statistical analysis of the results obtained for *CTNNB1 *mRNA expression revealed a significant effect of time across groups (P = 0.0002). Expression was induced in thoracic skin in response to wounding, with significantly elevated levels attained from one week on. The level of expression of *CTNNB1 *mRNA was significantly greater in thoracic than limb wounds from three weeks on; after six weeks of repair, RT-PCR showed a persistent induction of *CTTNB1 *in thoracic wounds.

**Figure 2:**
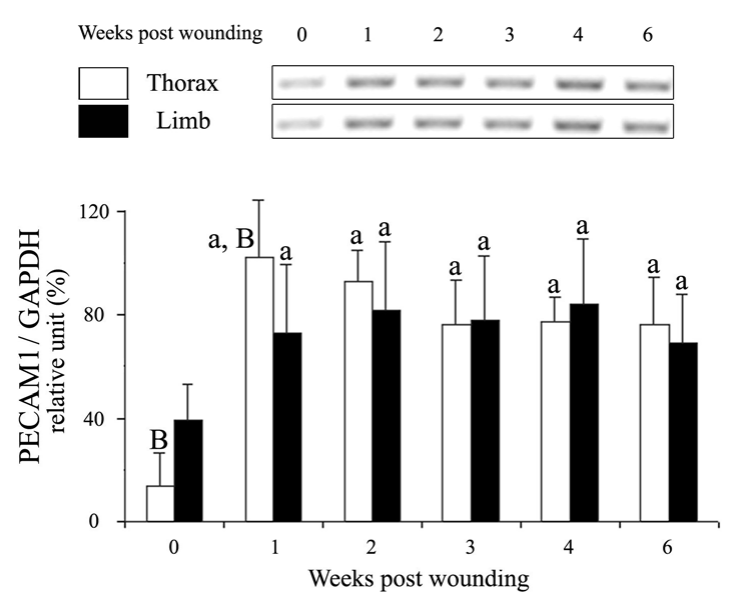
Regulation of equine *PECAM1 *mRNA by wounding of thorax and limb skin. Total RNA was extracted from wound margin biopsies isolated 1, 2, 3, 4 and 6 weeks post-wounding, then used in mRNA expression analyses by semi-quantitative RT-PCR as described in Methodology. Bar graphs represent the average of measures, performed in triplicate, on the mRNA of the four horses included in the study. Top: Regulation of *PECAM1 *mRNA [amplified fragment (AF) 548 bp] in wound biopsies from the thorax and the limb. Bottom: Relative changes in *PECAM1 *mRNA in biopsies of thorax and limb wounds. The intensity of *PECAM1 *signals was normalized with the control gene *GAPDH*. Different letters denote samples that differed significantly (P < 0.05) from time 0 of the same site (a); between anatomic sites at the same time (B). Data are presented as means ± SEM.

The repeated measures linear model revealed a non-significant effect of site, across time (P = 0.93), when used to analyze the temporal expression of *PECAM1 *mRNA during wound repair in the horse, though constitutive expression of *PECAM1 *in unwounded skin was significantly greater in the limb than in the thorax (P < 0.03; Fig 2). Thereafter the effect of site varied from one time to another. Semi-quantitative RT-PCR showed an induction of mRNA expression in response to wounding, with a significantly higher level reached in thoracic than in limb wounds after only one week of repair (P = 0.0174). *PECAM1 *mRNA expression did not return to baseline levels by the end of the 6-week study at either location. Indeed, the effect of time, across sites, was significant (P < 0.0001).

### Protein localization in healing body and limb wounds

**Figure 3:**
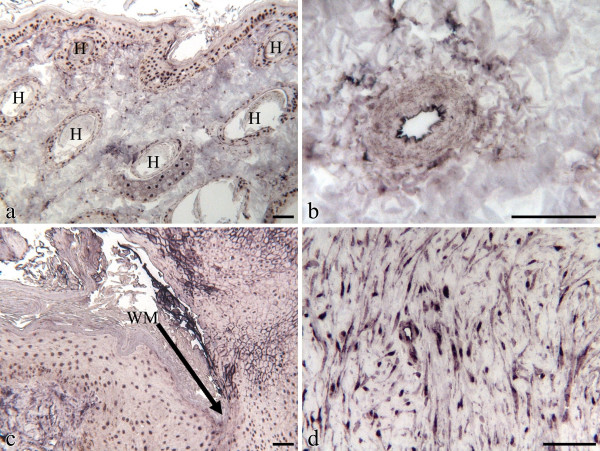
Immunohistochemical localization of β-catenin in the epidermal compartment of equine skin or wounds. Immunohistochemistry was performed on formalin-fixed, paraffin embedded tissues incubated with anti-β-catenin antibody as described in Methodology. Staining was absent when the primary antibody was omitted or substituted with normal serum (data not shown). The scale bar is equal to 0.1 mm. a) Unwounded limb skin. H = hair follicle. b) Unwounded limb skin. Endothelial cells stained with β-catenin antibody. c) Wound margin biopsy taken from 2 week old limb wound. WM = wound margin. d) 3 month old exuberant granulation tissue.

Immunoblotting showed a specific band of the expected molecular weight in equine skin protein extract for β-catenin determining that the commercial mouse monoclonal antibody raised against amino acids 680–781 mapping at the C-terminus of β-catenin of human origin (sc-7963; Santa Cruz Biotechnology) was specific for equine tissues (data not shown). Cellular localization of β-catenin in normal skin and wound tissues was determined by immunohistochemistry. In normal unwounded skin, the epidermis showed nuclear reactivity, which was also present in the sheaths of the hair follicles and some sebaceous glands (Fig 3a). No immunoreactive signal was noted in the extracellular matrix (ECM) or the fibroblasts populating the dermis, but endothelial cells were positively stained on their luminal side (Fig 3b). No qualitative or quantitative differences in staining were detected between thoracic and limb skin samples, nor later on between thoracic and limb wound samples. While epithelialization was incomplete two weeks following wounding, the intact epidermis adjacent to the wound continued to show nuclear reactivity but also new membrane reactivity at the wound edge (Fig 3c). The dermis contiguous to the wound showed the same pattern as pre-operatively while the granulation tissue filling the wound bed showed nuclear and cytoplasmic staining of both the fibroblasts and the microvascular endothelial cells. In the fourth week of repair the epidermis and the dermis showed the same staining pattern as described for the second week samples. The granulation tissue fibroblasts were strongly stained in a nuclear fashion while the endothelial cells were also labelled with the anti-β-catenin antibody. Six weeks following wounding, immunostaining for β-catenin protein had started to wane. The epidermis, which covered the wound bed in most cases, showed the nuclear staining pattern observed pre-operatively in intact skin. The granulation tissue, which had become less cellular, was weakly stained compared to that of four week biopsies and exhibited a staining pattern more nuclear than cytoplasmic. In three month and six month old EGT, fibroblasts as well as endothelial cells appeared strongly stained in a nuclear/cytoplasmic fashion (Fig 3d).

**Figure 4:**
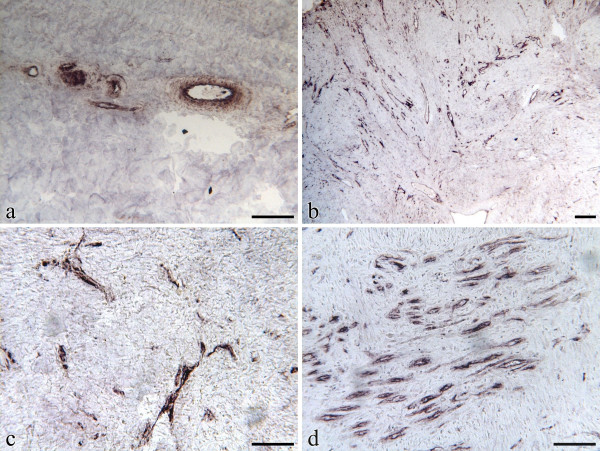
Immunohistochemical localization of PECAM1 in the dermal compartment of equine skin or wounds tissues. Immunohistochemistry was performed on formalin-fixed, paraffin embedded tissues incubated with anti-PECAM1 antibody as described in Methodology. The blood vessel endothelial cells are stained. Staining was absent when the primary antibody was omitted or substituted with normal rabbit serum (data not shown). The scale bar is equal to 0.2 mm. a) Unwounded thoracic skin. b) 6 month old exuberant granulation tissue. c) Wound margin biopsy (focusing on granulation tissue) taken from 4 week old healing thoracic wound. d) Wound margin biopsy (focusing on granulation tissue) taken from 4 week old healing limb wound.

The commercially available PECAM1 antibody (sc-1506; Santa Cruz Biotechnology) stained very specifically endothelial cell membranes, as expected. The PECAM1 antibody stained the luminal membrane of endothelial cells lining dermal blood vessels in normal, unwounded skin (Fig 4a) and in wound biopsies. Consequently, the staining pattern paralleled that of angiogenesis following wounding. At weeks 1 and 4 the presence of more blood vessels within the granulation tissue filling the wound bed translated into more PECAM1 immunostaining, particularly in limb wounds (Figs 4c and 4d). In 3 month old EGT, there were large and strongly PECAM1-positive vessels while in 6 month old EGT the vessels were smaller and more organized such that the PECAM1 staining was slightly weaker (Fig 4b).

## Discussion

The purpose of this study was to determine the temporal expression of CTNNB1/β-catenin and of PECAM1 during the repair of body and limb wounds in horses in an effort to define some of the molecular mechanisms leading to undue scarring. At the time of the study, the cDNAs were not characterized for the horse therefore both were cloned and sequenced. Hybridization of the size-selected libraries revealed the clones containing the full-length sequence of *PECAM1 *but only a truncated fragment of *CTNNB1*. Fortunately, the latter allowed the design of a homologous oligonucleotide that was used, along with a heterologous one, to amplify the full-length equine *CTNNB1 *cDNA sequence.

Results show equine *CTNNB1 *to be a highly conserved gene and to contain all 16 exons reported in humans. The excellent identity of the sequences for *CTNNB1 *in mammals (close to 100%) validates the quality of the equine sequence we obtained despite the required amplification by PCR. A high rate of degradation may have contributed to our inefficiency in obtaining the correct hybridization size for the full-length *CTNNB1 *gene in horse; indeed, in man there exists in the 3'-UTR one copy of AU-rich element (ATTTA), a motif known to contribute to short lived mRNAs.

*CTNNB1*, first cloned by McCrea et al. [27] in Xenopus laevis, shows no similarity in sequence to the genes for the α-catenins reported in other species while the β-catenin protein shares 70% amino acid identity with both plakoglobin, found in desmosomes, and the product of the Drosophila segment polarity gene "armadillo". Analysis of equine predicted β-catenin, against a protein conserved domains database (PROSITE), showed the presence of 9 Armadillo/plakoglobin repeat profiles, an approximately 40 amino acid long repeated sequence motif. Proteins that contain this type of domain combine structural roles and signaling functions, by generating and transducing signals affecting gene expression [28]. The calculated expected molecular weight (85,5 kDa) concords with that previously reported for other species.

A preliminary mRNA gene expression profiling study identified a cDNA fragment that corresponded to *PECAM1*, suggesting its up-regulation in response to dermal wounding [4]. The equine sequence determined herein contained the 16 exons present in the full-length human *PECAM1*, the predominant isoform detected in human tissue and endothelial cells [26]. This contrasts with the main isoform found in murine endothelium which lacks exons 14 and 15 [29]. While it is possible that other isoforms also exist in horses, our data suggest that, at least in skin, the full-length form predominates.

Processes occurring during the proliferative phase of wound repair ensure restoration of the structural and functional characteristics of skin which depend on both the epithelial and the mesenchymal components of the wound. The severity of scarring of horse limb wounds is related to an excessive proliferative phase where angiogenesis and fibroplasia are exacerbated [30-33]. This may result from dysregulation of the molecular components governing the growth of mesenchymal cells. In the study reported herein we have focused on two molecules, *CTNNB1 *and *PECAM1*, for their proposed role in endothelial cell and fibroblast proliferation.

The temporal patterns we report for mRNA expression of *CTNNB1 *and *PECAM1 *resemble one another. Both genes were constitutively expressed in normal intact skin, but while dermal trauma induced the expression of both mRNAs, the response was more acute for *PECAM1 *whose levels were significantly increased over baseline one week following wounding compared to two weeks for *CTNNB1*. This significant up-regulation of mRNA was maintained for the duration of the study for *PECAM1 *at both anatomic locations and for *CTNNB1 *in thoracic wounds; conversely, expression of *CTNNB1 *in limb wounds was not significantly elevated following wounding. Finally, mRNA expression in thoracic wounds significantly surpassed that of limb wounds for *PECAM1*, one week post-wounding, and for *CTNNB1*, three, four and six weeks post-wounding.

While possible that the temporal pattern of mRNA expression might reflect the different levels at the various sampling sites, the wounds were very close together such that we think it unlikely that anatomic or metabolic differences (blood supply, muscle coverage, etc) are important. Previous studies using a similar model [30-33] have not reported an influence of the exact location on the body or the lower limb. Likewise a current study has shown no difference in the rate of closure between wounds located distally or more proximally on the lower limb (Monteiro et al, unpublished data).

The correspondence in the patterns of mRNA expression following wounding is consistent with the PECAM1/β-catenin protein partnership proposed by Biswas et al. [7,34,35]. Indeed, the cytoplasmic portion of PECAM1 binds cytosolic β-catenin and this dynamic interaction is involved in angiogenesis [16]. More specifically, by enhancing the accumulation of stabilized β-catenin, PECAM1 modulates microvascular endothelial cell proliferation via transcriptional activation of growth promoting genes. Furthermore, a significant increase in *PECAM1 *mRNA levels preceded that of *CTNNB1 *following wounding in our study, consistent with the fact that upon cytokine stimulation endothelial cells initially induce PECAM1, followed by an increased expression of β-catenin [7].

While one might have anticipated higher levels of equine *CTNNB1 *mRNA in limb wounds predisposed to the development of EGT, a form of fibroproliferative disease, our opposite findings may reflect the mechanism of post-transcriptional regulation of β-catenin during wound repair, which controls how mRNA is translated into protein. Indeed, Cheon et al. documented that in the absence of any change in mRNA level, β-catenin protein, expressed primarily in dermal mesenchymal cells, was nonetheless transiently increased during the proliferative phase of normal wound healing in humans while exhibiting a sustained elevation in hyperplastic wounds [5]. Thus, a lack of correlation between mRNA and protein levels might underlie our unexpected findings; specifically, why *CTNNB1 *mRNA levels remained elevated when immunostaining for β-catenin waned, and why *CTNNB1 *mRNA levels in thoracic wounds surpassed those of limb wounds at weeks 3, 4 and 6 of healing.

In the horse, epithelial cells migrate slowly on limb wounds which are concurrently afflicted by over-abundant fibroblast proliferation; both are influences attributed to β-catenin protein [10,13]. Although immunohistochemical studies did not suggest quantitative differences in β-catenin expression between body and limb wounds, our investigation did reveal that while the protein remains transcriptionally active (nuclear stain) in dermal mesenchymal cells for the duration of the study, the intensity of the stain diminishes between the 4^th ^and 6^th ^weeks of healing. This pattern suggests that the signal would have disappeared entirely from normally healed wounds had the study been extended a few weeks. Conversely, strong immunostaining for β-catenin was detected in biopsies of 3 and 6 month old EGT, a condition characterized by exaggerated angiogenesis and fibroplasia and which resembles hyperplastic scarring in man in which a prolonged duration of β-catenin protein elevation has been reported [5]. Our findings may indicate that mesenchymal cells in EGT are behaving as though in a prolonged active proliferative phase of wound healing. In other words, the high levels of β-catenin would encourage proliferation and motility of the dermal cells, generating a larger dermal component to the wounds and hampering remodeling which requires reduced β-catenin levels [5].

While the design of our study cannot establish a cause-and-effect relationship between the prolonged elevation of β-catenin and the development of equine EGT, transgenic mouse models expressing a stabilized form of β-catenin in mesenchymal cells develop hyperplastic cutaneous wounds [11]. Fibroblasts derived from these mice display increased proliferation, motility and invasiveness when grafted into nude mice. Moreover, primary cell cultures demonstrate Tcf-dependent transcriptional activation [11] consistent with the hypothesis that nuclear β-catenin transactivation of target genes is a primary component of fibrosis.

In contrast to the membrane staining of epithelial cells reported by Cheon et al [5], we identified nuclear β-catenin within epithelial cells of intact skin. Our finding is similar to that of Tsuji et al. [36] who report nuclear immunostaining for β-catenin from the upper spinous to the granular cells in normal, intact human epidermis. The presence of stabilized, transcriptionally active β-catenin no doubt reflects keratinocyte proliferation and differentiation in response to continued renewal of the epithelial component of equine skin [37,38].

While PECAM1 is best known as a promoter of angiogenesis, its ability to facilitate neutrophil transendothelial migration [20,21] and thus boost the acute inflammatory response [39] is particularly intriguing in view of the data obtained herein. Indeed, it is tempting to draw a parallel between the inefficient and prolonged inflammatory response to wounding in the horse limb [33] and the significantly inferior expression of *PECAM1 *mRNA in wounds at this site compared to those of the thorax, one week following injury. Conversely, while differences in expression between the two sites disappear from the second week on, one might have expected an increase in limb wounds where new blood vessels are particularly abundant within the granulation tissue [33], in relation to PECAM1's role in angiogenesis. Interestingly, the expression pattern of PECAM1 isoforms has been shown to change during tube formation *in vitro*, indicating specialized roles for specific isoforms of PECAM1 during angiogenesis [26]. While our data suggest that we have cloned the predominant isoform of equine PECAM1, it is possible that another isoform is responsible for modulating endothelial cell adhesive properties during angiogenesis in the horse. Finally, the *PECAM1 *mRNA expression pattern found in our study corroborates previous assertions concerning the sluggishness hindering the repair of equine wounds, particularly those located on the extremities [1,30,33]. To this effect, a recent study in mice showed that both mRNA and protein expression of PECAM1 were induced three days following dermal wounding but mRNA expression returned to baseline by day 12 [40]. In contrast, expression of equine *PECAM1 *mRNA was significantly up-regulated in both thoracic and limb wounds for the 6-week duration of the study.

Immunohistochemistry revealed that, as expected of an endothelial cell marker, PECAM1 is principally present in blood vessels. As such, our data reflect that previously reported by Lepault et al. [33] who documented, histologically, more pronounced angiogenesis in healing limb than body wounds of horses. The concomitant presence of PECAM1 and β-catenin on endothelial cells of wound granulation tissue further supports the dynamic interaction between these two molecules, which may modulate the proliferation phase of angiogenesis [7,16].

## Conclusion

This study is the first to characterize equine cDNA for *CTNNB1 *and *PECAM1 *and to document that the genes are expressed over the different phases of wound repair in horses. Our findings suggest that β-catenin and PECAM1 might interact to modulate endothelial cell and fibroblast proliferation during wound repair in the horse.

Most previous studies investigating the roles of β-catenin and PECAM1 in wound healing have used *in vitro *techniques. Since wound repair is a complex process involving the interplay of several cell types, signaling pathways, extracellular matrix components and soluble factors, the role of various factors and their interactions may best be evaluated using an *in vivo *approach. Thus, while data interpretation can be challenging, the value of our study lies in the fact that the findings are more representative of what truly occurs in the patient.

In an effort to develop targeted therapies to prevent the formation of EGT leading to excessive scarring of equine limb wounds, future studies should quantitatively verify the temporal protein expression of β-catenin and PECAM1 and attempt to elucidate how the two molecules interact. The clues gained from studying the equine model may eventually lead to new insights into how to prevent unwanted scar formation in humans.

## Methods

### Cloning of equine *CTNNB1 *and *PECAM1*

Isolation of the full-length equine cDNAs was undertaken by screening size-selected cDNA libraries. The sizes of the full-length equine *PECAM1 *and *CTNNB1 *cDNA were estimated by virtual Northern blot analysis. Briefly, total RNA was isolated from a wound edge biopsy obtained 7 days following creation of a square 6.25 cm^2 ^full-thickness wound on the lateral thoracic wall [4] and transformed into cDNA by the SMART cDNA synthesis method (BD Biosciences Clontech, Mississauga ON) as described [41]. The cDNA was separated by gel electrophoresis, transferred onto a nylon membrane and hybridized with an equine radioactive probe (*CTNNB1 *= 535 bp: [GenBank:DN625863]; *PECAM1 *= 492 bp: [GenBank:DN625893]) generated from a previous gene expression profiling experiment [4]. On the basis of the hybridized size, a specific library was established via the pDrive plasmid cloning technique (Qiagen PCR cloning kit; Qiagen, Mississauga ON) and screened by radioactive hybridization as described [41]. Positive hybridizing bacterial colonies were grown, their plasmid contents were isolated (QIA-prep, Qiagen) and the size of the cloned cDNA was analyzed via gel electrophoresis analysis following *EcoR*1 digestion.

Since only a 3'-truncated cDNA fragment was obtained for *CTNNB1 *following library screening, a PCR reaction was performed to characterize 5' upstream sequences. Oligos were designed based on conserved regions of human [GenBank:NM_001904], swine [GenBank:NM_214367] and murine [GenBank:NM_007614] sequences. PCR reactions were performed using SMART cDNAs from a 7 day wound edge biopsy, Advantage 2 DNA polymerase (BD Biosciences Clontech) and PCR primers (sense: 5'-GCGTGGACAATGGCTACYCAAGC-3'; anti-sense: 5'-CCAGGCCAGCTGATTACTGTCAC-3'). The PCR product was cloned in the pDrive plasmid and produced as described above.

The cDNAs were sequenced via the dideoxy sequencing method (Big Dye Terminator 3.0; ABI Prism, Applied BioSystem, Branchburg NJ) and analyzed on an ABI Prism 310 sequencer (Applied BioSystem). Nucleic acid sequences were analyzed by BLAST and protein sequences deduced from cDNAs were analyzed by PSI- and PHI-BLAST [42] against GenBank data banks.

### Equine tissues and RNA extraction

Thoracic and limb normal skin as well as wound edge biopsies were taken at specific times during the repair process from four normal, 2-to-3 year old Standardbred mares, as described [33]. Briefly, five square 6.25 cm^2 ^areas were excised on the dorso-lateral aspect of one randomly assigned metacarpus beginning just above the fetlock, and on the lateral thoracic wall, 1.5 cm apart in a staggered vertical column, then left to heal by second intention. Excised skin from the lowermost wound was kept as a time 0 sample. One wound per site (thorax; limb) was sampled at the following times in each horse: 1, 2, 3, 4, and 6 weeks. To avoid repeated trauma, each wound, beginning with the most distal one, was designated for a single biopsy. Full-thickness specimens were taken with an 8 mm diameter biopsy punch to include a 3-to-4 mm strip of peripheral skin, the migrating epithelium and a 3-to-4 mm strip of granulation tissue from the wound center, when present. Biopsy samples were divided in half; one half was formalin-fixed and paraffin-embedded (FFPE) for immunohistochemical studies while the other was snap-frozen in liquid nitrogen and stored at -80°C until further processing. Total RNA from all samples was extracted and analyzed as previously described [43]. Samples of 3 and 6 month old EGT were obtained from two clinical cases presented to the Centre Hospitalier Universitaire Vétérinaire of the Université de Montréal and FFPE for immunohistochemistry. These experiments were approved by the Animal Ethics Committee of the Faculté de Médecine Vétérinaire of the Université de Montréal and were sanctioned by the Canadian Council on Animal Care.

### Semi-quantitative RT-PCR analyses of temporal expression of mRNA

Total RNA was first transformed into cDNA. One μg of total RNA from samples of healing thoracic wounds (n = 4 mares) was pooled for each biopsy time; the same was done for limb wound samples. From each of these pools, RNA was reverse-transcribed with an oligo-dT30 primer and PowerScript (BD Biosciences Clontech) to generate the first strand cDNA using the SMART PCR cDNA synthesis kit (User manual: PT3041-1; BD Biosciences Clontech) [44]. Second cDNA strands were produced and PCR-amplified using Advantage 2 DNA polymerase (BD Biosciences Clontech). To perform semi-quantitative RT-PCR, SMART cDNA pools were used in a 25 μl PCR reaction with the Advantage 2 DNA polymerase kit (BD Biosciences Clontech). Gene-specific PCR primers were designed in the open reading frame (ORF) of the equine cDNA sequence for: *CTNNB1 *(sense: 5'-GGACCACAAGCAGAGTATTGAAGG-3'; anti-sense: 5'-AATTCGGTTGTGAACATCCCGAGC-3'; [GenBank:DQ267491]), *PECAM1 *(sense: 5'-GGGACATATACCTGCACCGCA-3'; anti-sense: 5'-TTACTCGCCTGCGACTCATGC-3'; [GenBank:DQ310372]) and glyceraldehyde-3-phosphate dehydrogenase (*GAPDH*; sense: 5'-CAAGTTCCATGGCACAGTCACGG-3'; anti-sense: 5'-AAAGTGGTCGTTGAGGGCAATGC-3'; [GenBank:AF157626]) [4]. For all samples, PCR was performed in triplicate by Mastercycler^® ^ep. The number of cycles used was optimized for each gene to fall within the linear range of PCR amplification: *GAPDH *= 18 cycles; *CTNNB1 *= 23 cycles; *PECAM1 *= 22 cycles. The PCR reactions (20 μl/reaction) were resolved on a 2% TAE-agarose gel (40 mM Tris acetate; pH 8; 1 mM EDTA) with ethidium bromide (0.5 μg/ml); PCR products were visualized by UV and the images were digitized. The digitized signals for each gene were analyzed by densitometry using the NIH Image program [45].

### Immunoblot analyses

Specimens of equine skin and platelets were homogenized in M-PER buffer (Pierce, Rockford, IL) that was supplemented with a mix of protease inhibitors (Complete; Roche Applied Science, Laval, QC) as recommended by the manufacturer. Samples were homogenized at 7,000 rpm with a polytron PT1300D (Kinematica AG, Littau-Lucerne, Switzerland). The protein extracts were centrifuged at 10,000 × g for 5 min at 4°C, and the recovered supernatant (whole cell extract) was stored at -80°C until electrophoretic analyses were performed. Protein concentration was determined by the Bradford method [46] (Bio-Rad Protein assay, Bio-Rad Laboratories Inc., Hercules, CA). Protein extracts (100 μg proteins/sample) were heat-treated (5 min, 100°C) and size-fractionated via a one-dimensional SDS-PAGE, then electrophoretically transferred onto polyvinylidene difluoride membranes (PVDF; Hybond-P, Amersham Pharmacia Biotech). Immunoblots were performed as described [47]. Membranes were incubated either with the mouse anti-human β-catenin monoclonal antibody (dilution 1:1,000; sc-7963; Santa Cruz Biotechnology, Inc.) or with the goat anti-mouse PECAM-1 polyclonal antibody (dilution 1:200; sc-1506; Santa Cruz Biotechnology, Inc. Santa Cruz, CA). As secondary antibodies a sheep anti-mouse IgG-HRP (dilution 1:10,000; NA931; Amersham Pharmacia Biotech) and a donkey anti-goat IgG-HRP (dilution 1:5,000; sc-2020; Santa Cruz Biotechnology, Inc.) were used. Detection of immunoreactive proteins was performed by the enhanced chemiluminescence system (ECL Plus, Amersham Pharmacia Biotech) following the manufacturer's protocol, and exposed to Hyperfilm (Amersham Pharmacia Biotech). Autoradiographic images were digitized using a ScanMaker 9800XL flatbed scanner (Microtek lab, Inc., Redondo Beach CA).

### Immunohistochemical localization of β-catenin and *PECAM1*

PBS-buffered formalin-fixed tissues from both locations (thorax and limb) in all four horses were prepared as described [47]. Paraffin-embedded tissues were cut to 3 μm thickness, mounted on SuperfrostPlus slides (Fisher Scientific, Pittsburgh PA), deparaffinized and then rehydrated. Antigenicity lost during the fixation process was retrieved by pressure cooker heat treatment for 15 min [47]. Non-specific binding sites were saturated by 30 min incubation in blocking buffer: TBS (100 mM Tris; pH 7.5; 150 mM NaCl), 1% bovine serum albumin and 1% fat-free skim milk. For the antibody raised in goat, the fat-free skim milk was omitted; the saturation step was substituted by 30 min incubation in TBS-tween (100 mM Tris; pH 7.5; Tween 0.2%) Tissue sections were incubated overnight at 4°C with the primary antibodies (β-catenin 1:25; PECAM1 1:50) diluted in blocking buffer. Negative control tissue sections were incubated similarly. After three 10 min washes in TBS, complexes were detected by incubation for 2 h at room temperature with an anti-goat/mouse IgG alkaline phosphatase conjugated (Sigma-Aldrich) diluted to 1:100 in blocking buffer. Tissue sections were washed twice in TBS and once in TBS-MgCl_2 _(100 mM Tris pH 9.5, 50 mM MgCl_2_) and incubated with the NBT/BCIP alkaline phosphatase substrate (Roche Applied Science). Sections were mounted in Vectamount^® ^Permanent Mounting Medium (Vector Laboratories, Burlingame CA). Photographs were taken under bright field illumination using a Nikon Eclipse E400 microscope equipped with a digital camera (Nikon Coolpix 4500). Digital images were processed and assembled by Photoshop software (Adobe Systems Inc., San Jose CA).

### Statistical analysis

Gene-specific signals (*CTNNB1; PECAM1*) were normalized with corresponding *GAPDH *signals for each sample. A repeated-measures (RM) linear model, with site (thorax vs limb) and biopsy time as within-subject factors, was used to determine the effects of site and time on gene expression. When the RM linear model indicated significant differences (P < 0.05), a priori contrasts were used to compare pre-selected individual means. All analyses were carried out with a P value < 0.05, using SAS v. 9.1. (Cary, N.C.).

## Authors' contributions

VM, ZI and JLL were responsible for generating the majority of the data, including analysis of the wound healing experiments: ZI was responsible for the cloning; VM and JLL contributed to the cloning and carried out the immunoblotting and the immunohistochemistry; VM helped to draft the manuscript.

JGL and CLT were the senior investigators who conceived the study and participated in its design and coordination; CLT drafted the manuscript and JGL helped in the drafting.

All authors read and approved the final manuscript.
